# Supplementary material for: Anti-microRNA-1976 as a Novel Approach to Enhance Chemosensitivity in XAF1+ Pancreatic and Liver Cancer
Source: Biomedicines. 2023 Apr 10;11(4):1136. doi: 10.3390/biomedicines11041136 (PMC10135778; doi:10.3390/biomedicines11041136)
Supplement: Supplementary file 1 [file biomedicines-11-01136-s001.zip › biomedicines-2216813-SM.pdf]

Supplementary Materials

Anti-microRNA-1976 as a Novel Approach to Enhance Chemosensitivity in *XAF1*⁺ Pancreatic and Liver Cancer

Tsai-Yen Lee, Chien-Jen Tseng, Jin-Wun Wang, Ching-Po Wu, Chin-Yuan Chung, Ting-Ting Tseng and Shao-Chen Lee

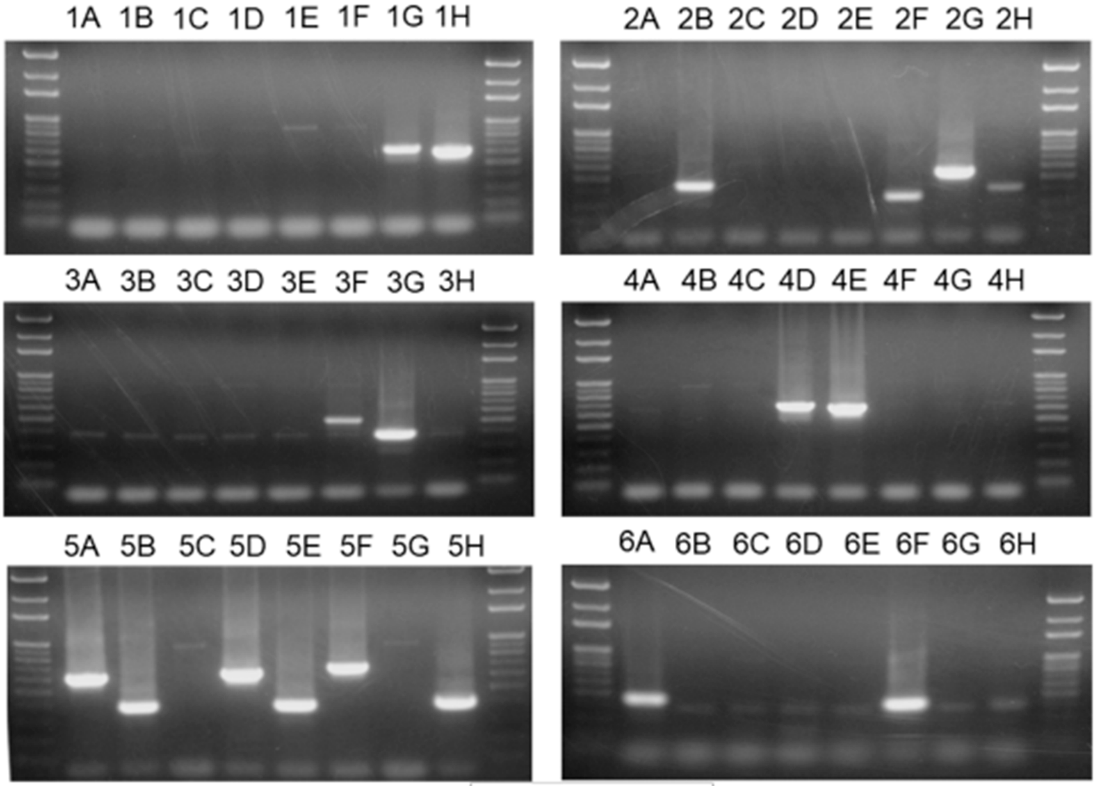

(**a**)

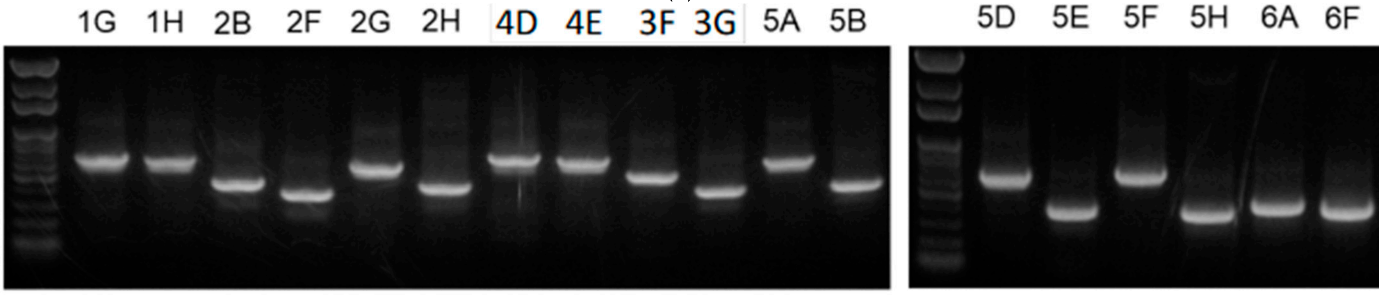

(**b**)

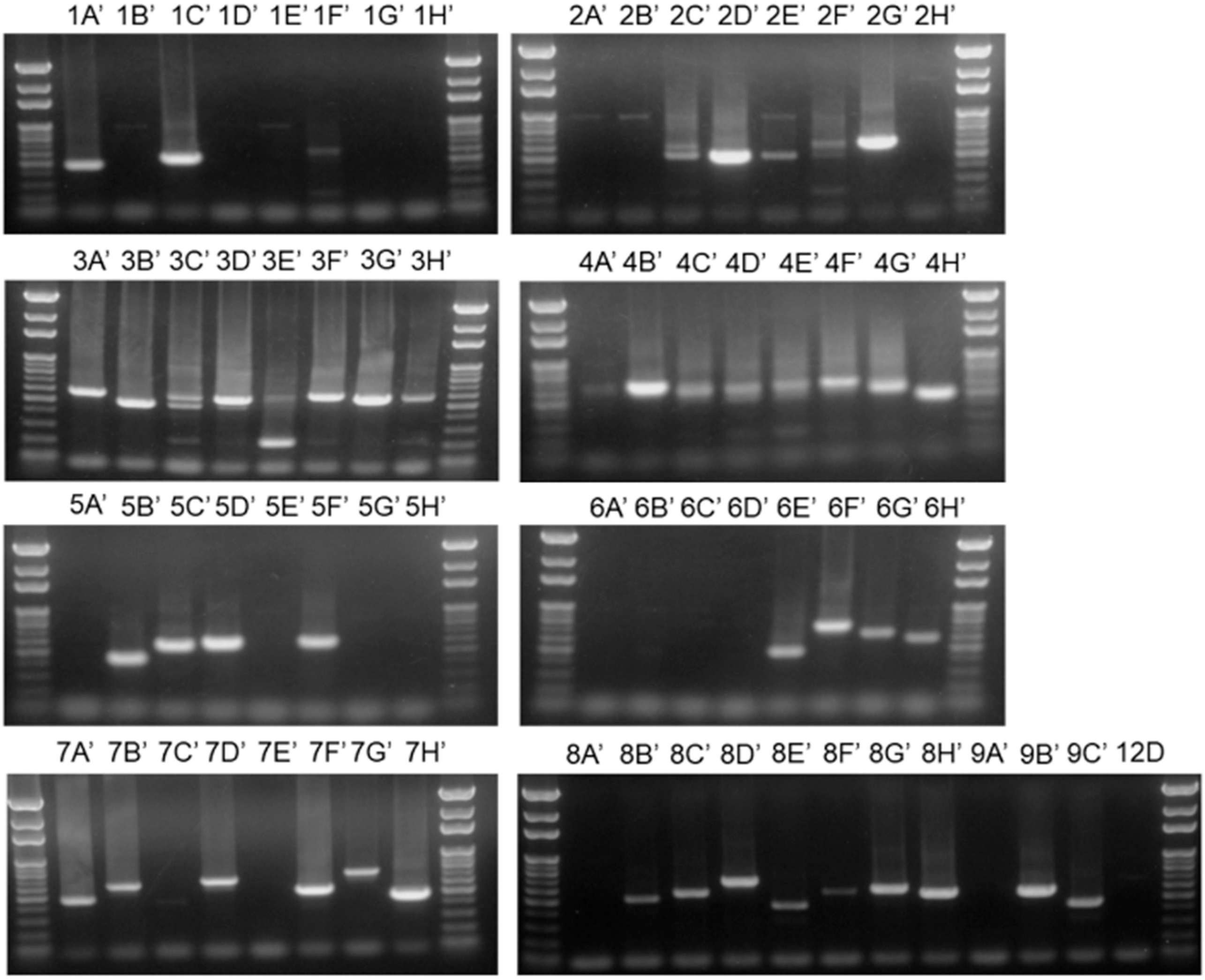

(**c**)

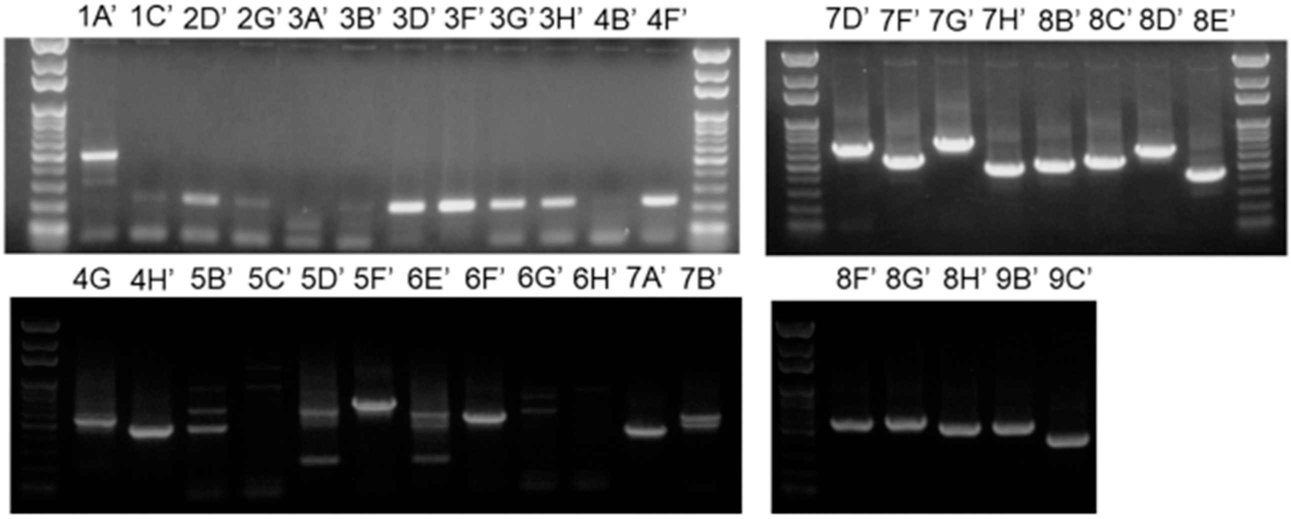

(**d**)

Figure S1. Colony PCR selection and validation of miR-1976 binding targets generated by miR-1976-specific cDNA cloning in Hep3B cells and HepG2 cells. The 48 selected clones (**a**) and 18 confirmed clones (**b**) in Hep3B cells were examined by PCR. The 68 selected clones (**c**) and 24 confirmed clones (**d**) in HepG2 cells were examined by PCR.

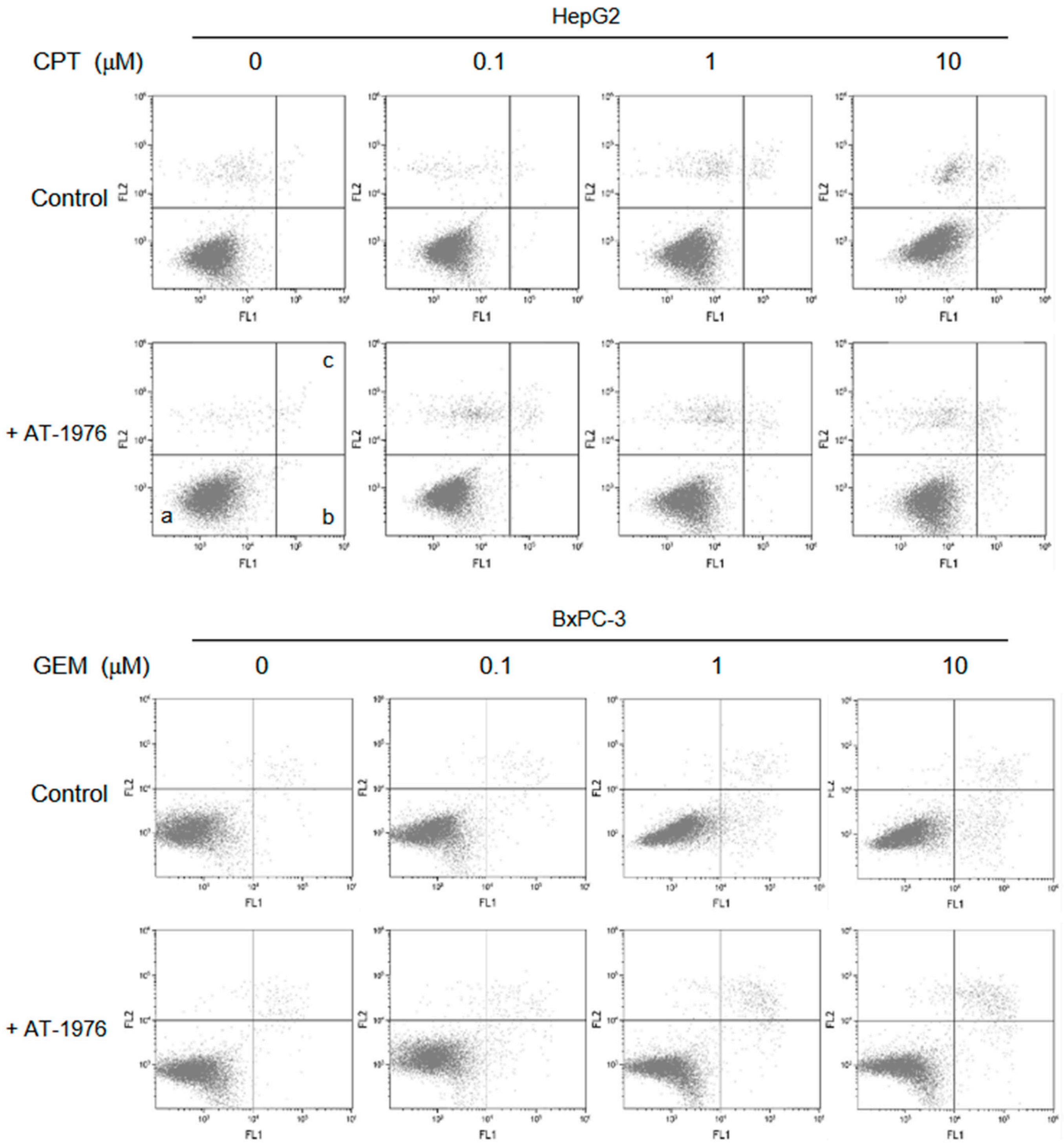

Figure S2. Cell apoptosis as examined by annexin V–PI staining and analyzed by flow cytometry. FITC–annexin V was detected and reported along FL1 (*x*-axis). PI was detected and reported at FL2 (*y*-axis). The percentages of cell populations in section a (healthy), section b (early apoptosis; Annexin V+PI-), and section c (late apoptosis; Annexin V+PI+) were counted and shown in Figure 5E.

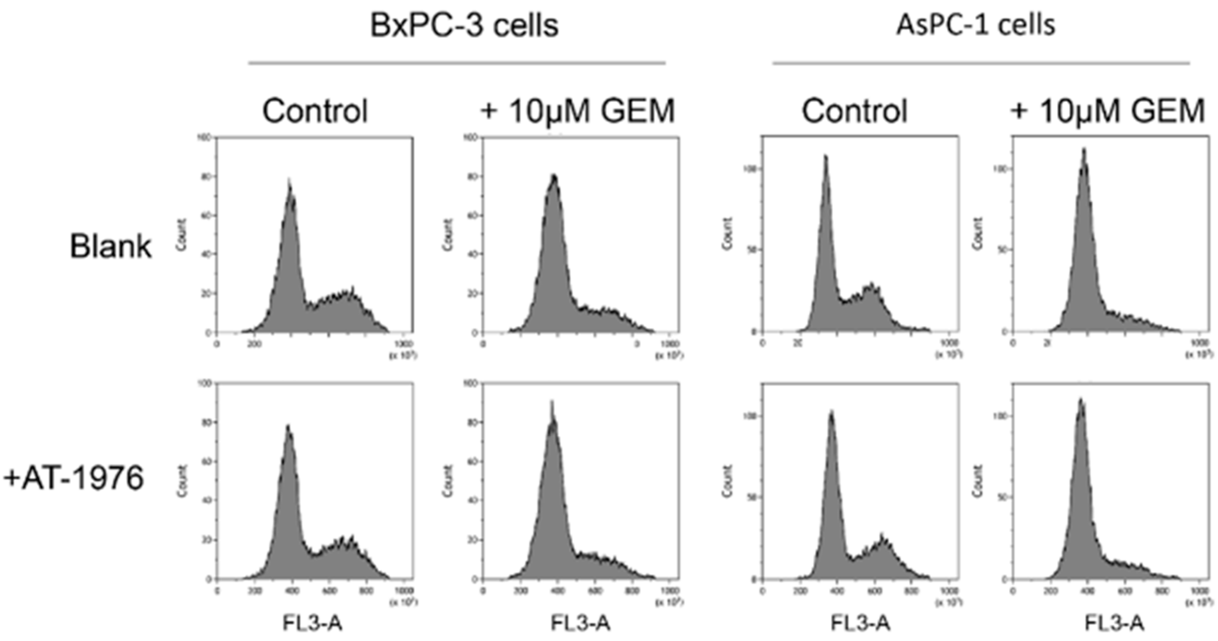

Figure S3. Cell cycle analysis of BxPC-3 (*XAF1*+) and AsPC-1 (*XAF1*-) cells with or without AT-1976 treatment. GEM treatment reduced G2 content, while AT-1976 co-treatment did not alter the profile of cell cycle populations, regardless of the presence of GEM.

Table S1. miR-1976 binding targets identified from sequencing results of gene-specific cDNA clones from Hep3B cells.

**Clone ID**	**Gene Name Sequence ID (identity %)**	**Duplex structure for miRNA target site**	**Folding energy (Kcal/mol)**
**3B_1G (forward) (reverse)**	Homo sapiens chromosome 1， alternate assembly CHM1_1.1; NC 018912.2 (100%) PREDICTED: Homo sapiens zinc finger protein 644 (***ZNF644***); XM 017002492.1; XM 011542260.2 (100%)	No result	
**3B_1H (reverse)**	Homo sapiens chromosome 3， alternate assembly CHM1_1.1; NC 018914.2 (99%)	No confirmed gene	
**3B_2F (forward)**	PREDICTED: Homo sapiens leukocyte receptor cluster (LRC) member 8m (***LENG8***) variant 1-8; XM 005278248.4 (100%)	GAAGGAAGGGGAGGCAGGAGG<br>|| ||||:| ||||||||| TGTCGTTCCTC-CCGTCCTCC	-29.90
**3B_2G (reverse)**	Homo sapiens chromosome 7， alternate assembly CHM1_1.1; NC 018918.2 (100%)	No confirmed gene	
**3B_2H (reverse)**	Homo sapiens chromosome 8， alternate assembly CHM1_1.1; NC 018919.2 (100%)	No confirmed gene	
**3B_3G (forward)**	Homo sapiens ankyrin repeat domain 17 (***ANKRD17***) variant 1-4; NM 0.32217.4; NM 198889.2;	ACAGT-TGGCAGGGCTGGGGC<br>||||: || ||||| ||:| TGTCGTTCC-TCCCGTCCTCC	-22.80

<table>
<tr><td></td><td>NM 001286771.2; NM 015574.1 (100%)</td><td></td><td></td></tr>
<tr><td>3B_4D (forward)</td><td>Homo sapiens G-patch domain containing 4 (GPATCH4)
NM 015590.3; NM 182679.2 (99%)</td><td>GCAGCAACAGGAGG--AGGAGG
:||||| |||||| ||||||
TGTCGT—TCCTCCCGTCCTCC</td><td>-26.90</td></tr>
<tr><td>3B_4E (forward)</td><td>Homo sapiens importin 8 (IPO8)
NM 006390.3; NM 001190995.1 (100%)</td><td>CTGGT-CACTGGCCAGGAGT
:|: || ||||||
TGTCGTTCCTCCCGTCCTCC</td><td>-12.00</td></tr>
<tr><td>3B_5A (forward)</td><td>PREDICTED: Homo sapiens kinectin 1 (KTN1),
Variant 2-3; NM 001079521.1 NM_001079522.1(99%)</td><td>No result</td><td></td></tr>
<tr><td>3B_5D (forward)</td><td>Homo sapiens importin 8 (IPO8) variant1-2;
NM 006390.3; NM 001190995.1 (100%)</td><td>CTGGT-CACTGGCCAGGAGT
:|: || ||||||
TGTCGTTCCTCCCGTCCTCC</td><td>-12.00</td></tr>
<tr><td>3B_6A (forward)</td><td>Homo sapiens tripeptidyl peptidase 1 (TPP1);
NM 000391.3 (99%)</td><td>CCTGTAA--CAGGCTGGGGA
|:|| ||| ||:|
TGTCGTTCCTCCCGTCCTCC</td><td>-9.80</td></tr>
<tr><td>3B_6F (forward)</td><td>Homo sapiens chromosome 1， alternate assembly
CHM1_1.1; NC 018912.2 (100%)</td><td>No confirmed gene</td><td></td></tr>
</table>

Table S2. miR-1976 binding targets identified from sequencing results of gene-specific cDNA clones from HepG2 cells.

<table>
<tr><th>Clone ID</th><th>Gene Name Sequence ID (identity %)</th><th>Duplex structure for miRNA target site</th><th>Folding energy (Kcal/mol)</th></tr>
<tr><td>G2_4H (reverse)</td><td>Homo sapiens chromosome 12, alternate assembly
CHM1_1.1; NC 018923.2 (99%)</td><td>No confirmed gene</td><td></td></tr>
<tr><td>G2_7A (reverse)</td><td>Homo sapiens chromosome 3, alternate assembly
CHM1_1.1; NC 018923.2 (99%)</td><td>No confirmed gene</td><td></td></tr>
<tr><td>G2_7H (reverse)</td><td>Homo sapiens tripeptidyl peptidase 1 (TPP1);
NM 000391.3 (99%)</td><td>CCTGTAA--CAGGCTGGGGA
|:|| ||| ||:|
TGTCGTTCCTCCCGTCCTCC</td><td>-9.80</td></tr>
<tr><td>G2_8B (reverse)</td><td>Homo sapiens chromosome 16, alternate assembly
CHM1_1.1; NC 018927.2 (99%)</td><td>No confirmed gene</td><td></td></tr>
<tr><td>G2_8C (reverse)</td><td>Homo sapiens solute carrier family 20 member 2
(SLC20A2) variant 1-3; NM 001257180.1; NM 006749.4; NM 001257181.1 (98%)</td><td>ACGGGACCGAGGGCAGGAGG
||:| | ||||||||||||
TGTCGTTCCTCCCGTCCTCC</td><td>-30.00</td></tr>
<tr><td>G2_8E (reverse)</td><td>Homo sapiens ribosomal protein L27 (RPL27)
NM 000988.3 (99%)</td><td>No result</td><td></td></tr>
<tr><td>G2_8F (forward)</td><td>Homo sapiens XIAP associated factor 1 (XAF1) variant
1-5; NM 017523.3; NM 199139.2 (99%)</td><td>ACAGCAA-TCAGGCAAGAGA
||||||| |||| |||
TGTCGTTCCTCCCGTCCTCC</td><td>-17.00</td></tr>
</table>

**G2_8H (forward)**	Homo sapiens WD repeat domain 5 (***WDR5***); NM 017588.2; NM 052821.3 (90%)	GAGGTCCTCGAGTGGCAGGGGT<br>:|: ||| ||||||:| TGTCG-TTCCTC-CCGTCCTCC	-18.40
**G2_9B (forward)**	Homo sapiens SEC62 homolog, preprotein translocation factor (***SEC62***); NM 003262.3 (100%)	ATGGC--GGAACGCAGGAGA<br>|::|| ||| ||||||| TGTCGTTCCTCCCGTCCTCC	-18.20
